# Adopting telehealth for mental health support in stroke rehabilitation in Sub-Saharan Africa: A conceptual analysis using Rogers’ Diffusion of Innovation Theory

**DOI:** 10.1371/journal.pgph.0005025

**Published:** 2026-02-05

**Authors:** Delight Tsogbe, Tettey Martin, Akuffo Bernice Gyanmea

**Affiliations:** Department of Population and Behavioral Sciences, Fred N. Binka School of Public Health, University of Health and Allied Sciences, Ho, Ghana; African Population and Health Research Center, KENYA

## Abstract

Stroke rehabilitation in Sub-Saharan Africa (SSA) faces persistent gaps in mental health service integration. Post-stroke depression, anxiety, and cognitive impairments remain underdiagnosed due to workforce shortages, stigma, and infrastructural constraints. Telehealth technologies offer a potential mechanism to expand access to psychological care, yet adoption across SSA remains uneven. This paper provides a conceptual, theory-driven synthesis exploring telehealth adoption for mental health support in stroke rehabilitation across SSA, guided by Rogers’ Diffusion of Innovation Theory. A structured conceptual review was conducted using literature from PubMed, Scopus, AJOL, and Google Scholar (2005–2024). Inclusion criteria focused on SSA studies addressing telehealth or mHealth in mental health or rehabilitation contexts. Thematic synthesis was guided by Rogers’ five innovation attributes, relative advantage, compatibility, complexity, trialability, and observability, to assess adoption facilitators and barriers. Telehealth adoption is influenced by cultural compatibility, simplicity of design, perceived relative advantage, observable benefits through pilot projects, and supportive policy environments. Successful initiatives in Ghana, Kenya, Nigeria, and Uganda demonstrate the importance of localized adaptation and user engagement. Telehealth provides a scalable pathway to address post-stroke mental health needs in SSA. Effective adoption requires culturally responsive design, targeted capacity-building, and policy alignment with national and regional digital health strategies.

## Introduction

Stroke remains one of the leading causes of disability and mortality in Sub-Saharan Africa (SSA). The mental health sequelae of stroke, such as depression, anxiety, and cognitive impairment, significantly reduce recovery outcomes. However, mental health services are severely limited due to infrastructural barriers, insufficient specialists, and sociocultural stigma [1,2].

Telehealth, encompassing video consultations, mobile health (mHealth), and remote rehabilitation, has emerged as a potential solution to overcome geographic and workforce barriers [3]. Despite increasing attention, telehealth adoption in SSA remains inconsistent, constrained by cost, digital literacy, and fragmented policy environments [4].

To understand these challenges conceptually, this paper applies Rogers’ Diffusion of Innovation Theory [5] to synthesize evidence on telehealth adoption for mental health support in stroke rehabilitation across SSA.

## Methods

### Conceptual design

This study adopts a theory-driven conceptual synthesis approach using Rogers’ Diffusion of Innovation Theory as the analytical framework. The approach integrates existing empirical and theoretical evidence to derive implementation insights rather than generating primary data.

### Theoretical framework

**Fig 1:**
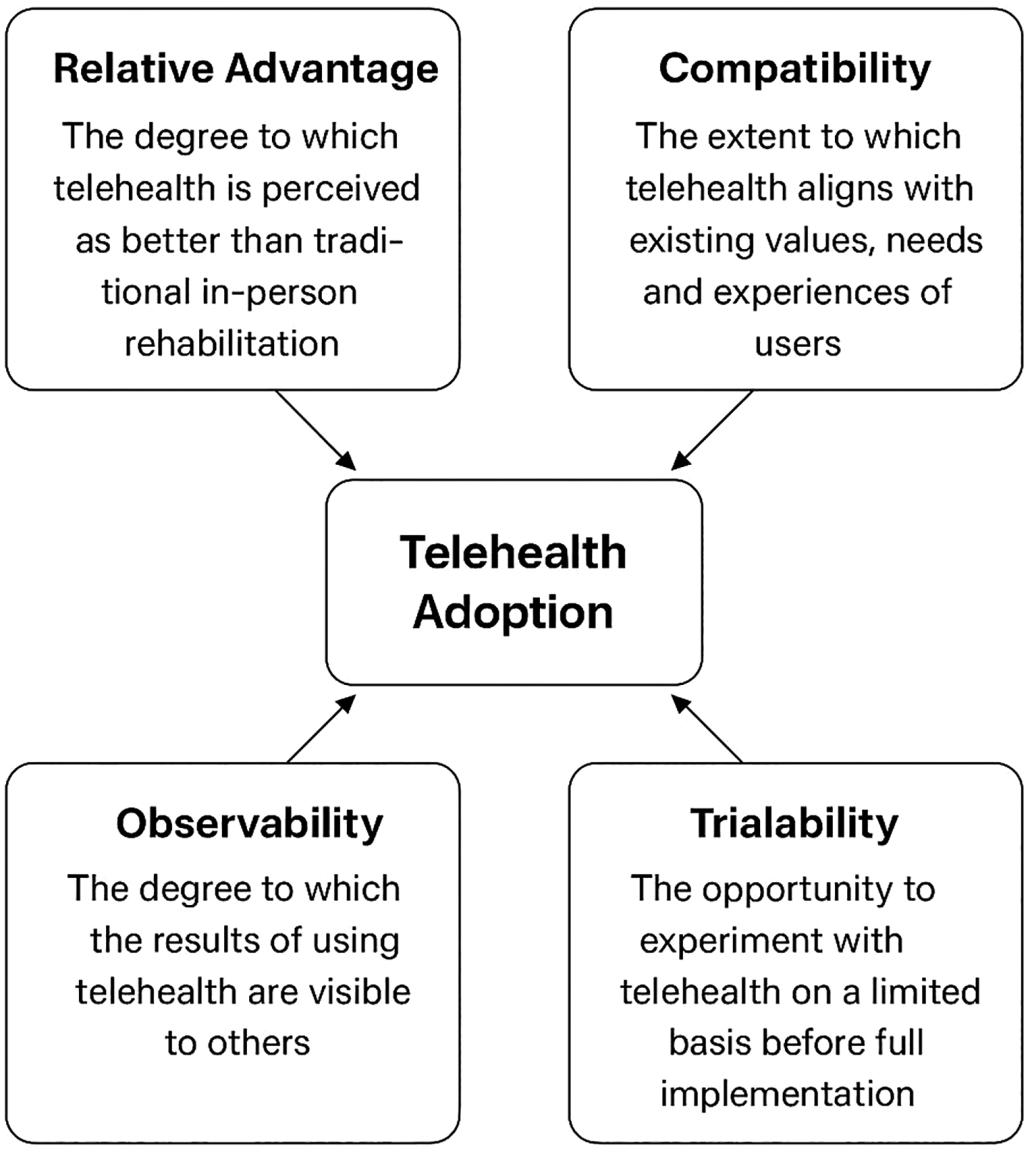
The five innovation attributes influencing telehealth adoption in stroke rehabilitation (adapted from Rogers, 2003).

Rogers’ Diffusion of Innovation Theory provides a systematic lens for understanding how new technologies, such as telehealth, are adopted within health systems. The theory categorizes adopters into five groups, innovators, early adopters, early majority, late majority, and laggards, each differing in their readiness to embrace innovation. It also identifies five key innovation attributes that influence adoption decisions: relative advantage (the perceived improvement over existing methods), compatibility (alignment with users’ values and needs), complexity (ease of use), trialability (the ability to experiment on a limited basis), and observability (visibility of benefits and outcomes). Applying this framework offers a structured understanding of how telehealth can be effectively adopted to provide mental health support during stroke rehabilitation across Sub-Saharan Africa, highlighting the social, technological, and contextual factors that shape diffusion dynamics. A schematic representation of these five innovation attributes and their relationship to telehealth adoption in stroke rehabilitation is illustrated in Fig 1.

### Search strategy

Databases searched included PubMed, Scopus, AJOL, and Google Scholar between 2005 and 2024. Search terms included:

“telehealth”, “mental health”, “stroke rehabilitation”, “Sub-Saharan Africa”, “digital health”, and “Diffusion of Innovation Theory”.

### Inclusion criteria

- Focus on Sub-Saharan Africa
- Telehealth or mHealth interventions for mental health or stroke rehabilitation
- Articles discussing implementation, barriers, or adoption outcomes
- Peer-reviewed or credible institutional reports

### Exclusion criteria

- Non-SSA contexts
- Studies unrelated to telehealth or mental health
- Commentary pieces lacking implementation evidence

### Data extraction and synthesis

Eligible studies were screened manually and coded under Rogers’ five innovation attributes. Key data extracted included country, intervention type, health condition, findings, and coded attribute. Reporting transparency followed ENTREQ and PRISMA-ScR recommendations. A summary of the reviewed studies across Sub-Saharan Africa is presented in Table 1.

**Table 1:** Summary of Reviewed Studies (2005–2024).

Country	Intervention Type	Focus Area	Key Findings	Innovation Attribute
Ghana	Telepsychiatry pilot (mHealth)	Post-stroke depression	Improved follow-up and access	Compatibility
Kenya	Mobile rehab platform	Stroke rehabilitation continuity	Feasible, low dropout rate	Trialability
Nigeria	Video-based teletherapy	Mental health counselling	Effective but limited by bandwidth	Complexity
South Africa	Hybrid telehealth for chronic care	Cognitive rehab post-stroke	Improved adherence	Relative Advantage
Uganda	SMS mental health support	Depression screening	Enhanced patient engagement	Observability

## Results and discussion

### Compatibility

Telehealth initiatives aligning with local clinical routines and cultural contexts show better uptake. Culturally adapted interventions, such as Ghana’s community telepsychiatry model, demonstrated higher engagement due to language inclusivity and integration with existing health infrastructure [6]. Lack of alignment with cultural norms or poor stakeholder involvement often hinder scalability [7].

### Complexity

Adoption depends heavily on technology usability and digital literacy. Simplified platforms with training modules have succeeded in Kenya and Ethiopia [8]. Conversely, high system complexity, unstable connectivity, and English-only interfaces create barriers for rural populations [9].

### Relative advantage

Perceived benefits, such as reduced travel costs, improved access to specialists, and enhanced continuity of care, drive adoption. Evidence from South Africa and Nigeria indicates that telehealth can complement face-to-face models by reducing logistical burdens [10,11].

### Trialability and observability

Pilot programs in Ghana and Uganda improved stakeholder trust by allowing experimentation in controlled settings. Observable benefits, such as improved therapy attendance and patient satisfaction, further encouraged diffusion [12]. Documented visibility of success is a strong determinant of scaling efforts across SSA [13].

### Critical appraisal of evidence

Most studies reviewed were small-scale or pilot interventions, limiting generalizability. Few reported long-term outcomes or cost-effectiveness analyses. The evidence base remains fragmented, highlighting the need for empirical studies that measure clinical outcomes, sustainability, and economic viability.

### Equity and ethical considerations

Digital health adoption in SSA must address digital exclusion, particularly among rural women, the elderly, and low-literacy groups [14]. Ethical concerns around data privacy, informed consent, and trust are also significant, given limited digital governance structures. Culturally sensitive design and strong data protection frameworks are essential for equitable telehealth expansion.

### Policy and implementation recommendations

- Integrate telehealth within national eHealth and UHC frameworks.
- Establish digital literacy training for health workers and patients.
- Support pilot-to-scale pathways through public-private partnerships.
- Ensure affordable digital infrastructure and regulatory oversight.
- Align strategies with African Union’s Digital Health Strategy (2023).

## Conclusion

Telehealth represents a transformative opportunity to strengthen post-stroke mental health rehabilitation in SSA. Adoption success depends on alignment with cultural and infrastructural realities, simplicity of design, visible benefits, and supportive policy ecosystems. A systems-level, equity-focused approach can ensure that telehealth advances inclusion rather than exacerbating digital divides.

## Limitations

This conceptual synthesis draws on secondary literature and does not include primary data collection. Findings depend on the quality and availability of existing evidence, which varies across SSA contexts.

## References

[1] Adjorlolo S, Ellingsen G. Readiness assessment for implementation of electronic patient record in Ghana: a case of university of Ghana hospital. Journal of Health Informatics in Developing Countries. 2013;7(2).

[2] Hackett ML, Köhler S, O’Brien JT, Mead GE. Neuropsychiatric outcomes of stroke. Lancet Neurol. 2014;13(5):525–34. doi: 10.1016/S1474-4422(14)70016-X 24685278

[3] Kruse CS, Krowski N, Rodriguez B, Tran L, Vela J, Brooks M. Telehealth and patient satisfaction: a systematic review and narrative analysis. BMJ Open. 2017;7(8):e016242. doi: 10.1136/bmjopen-2017-016242 28775188 PMC5629741

[4] Fatehi F, Samadbeik M, Kazemi A. What is digital health? Review of definitions. Integrated citizen centered digital health and social care. IOS Press. 2020. p. 67–71. 10.3233/SHTI200696 33227742

[5] Rogers EM. Diffusion of Innovations. 5th ed. New York: Free Press; 2003.

[6] Keteku-Atiemo WI. An Evaluation Of A Telehealth Intervention For A Chronic Care Group In Accra, Ghana (Doctoral dissertation, University of Ghana).

[7] Manyazewal T, Woldeamanuel Y, Blumberg HM, Fekadu A, Marconi VC. The potential of digital health technologies in African context, Ethiopia. medRxiv. 2021 Mar 29:2021-03. 10.1038/s41746-021-00487-4 PMC8371011 34404895

[8] Dodoo JE, Al-Samarraie H, Alzahrani AI. Telemedicine use in Sub-Saharan Africa: Barriers and policy recommendations for Covid-19 and beyond. Int J Med Inform. 2021;151:104467. doi: 10.1016/j.ijmedinf.2021.104467 33915421 PMC9761083

[9] Aderinto N, Olatunji G, Kokori E, Babalola AE, Ogieuhi IJ, Aboje JE, et al. Telepsychiatry in Africa: overcoming barriers to access and care. Discov Ment Health. 2025;5(1):64. doi: 10.1007/s44192-025-00197-3 40299230 PMC12040767

[10] Agbeyangi AO, Lukose JM. Telemedicine Adoption and Prospects in Sub-Sahara Africa: A Systematic Review with a Focus on South Africa, Kenya, and Nigeria. Healthcare (Basel). 2025;13(7):762. doi: 10.3390/healthcare13070762 40218059 PMC11989057

[11] Dodoo JE, Al-Samarraie H, Alsswey A. The development of telemedicine programs in Sub-Saharan Africa: Progress and associated challenges. Health Technol (Berl). 2022;12(1):33–46. doi: 10.1007/s12553-021-00626-7 34849325 PMC8613515

[12] WHO Regional Office for Africa. Digital Health in the WHO African Region: Progress and Opportunities. Brazzaville: WHO; 2023.

[13] Greenhalgh T, Robert G, Macfarlane F, Bate P, Kyriakidou O. Diffusion of innovations in service organizations: systematic review and recommendations. Milbank Q. 2004;82(4):581–629. doi: 10.1111/j.0887-378X.2004.00325.x 15595944 PMC2690184

[14] Udenigwe O, Yaya S. Leaving no woman or girl behind? Inclusion and participation in digital maternal health programs in sub-Saharan Africa. Reprod Health. 2022;19(1):54. doi: 10.1186/s12978-022-01358-1 35227260 PMC8884409

