# Supplementary material for: Adopting telehealth for mental health support in stroke rehabilitation in Sub-Saharan Africa: A conceptual analysis using Rogers’ Diffusion of Innovation Theory
Source: PLOS Glob Public Health. 2026 Feb 5;6(2):e0005025. doi: 10.1371/journal.pgph.0005025 (PMC12875508; doi:10.1371/journal.pgph.0005025)
Supplement: Attachment — Submitted filename: Response to Reviewers.docx [file pgph.0005025.s002.docx]

**Response to Reviewers**

**Manuscript ID:** PGPH-D-25-02080

**Title:** *Adopting Telehealth for Mental Health Support in Stroke Rehabilitation in Sub-Saharan Africa: A Conceptual Analysis Using Rogers’ Diffusion of Innovation Theory*

**Journal:** *PLOS Global Public Health*

Dear Prof. Razak Gyasi and Esteemed Reviewers,

We sincerely thank you for your thoughtful and constructive feedback on our manuscript. We have revised the paper extensively to address all the comments, improve methodological transparency, and align more closely with *PLOS Global Public Health*’s standards of rigor, clarity, and equity focus.

Below, we provide a point-by-point response to each comment and explain the corresponding revisions made in the manuscript.

**Reviewer #1**

**Comment 1:** The manuscript presents an interesting conceptual synthesis but needs to clearly distinguish itself from empirical research.

**Response:** We have clarified throughout the Abstract, Introduction, and Methods that this work is a *conceptual, theory-driven synthesis* guided by Rogers’ Diffusion of Innovation Theory. This distinction is emphasized in the Abstract (lines 5–9) and Methods (lines 80–90).

**Comment 2:** The methodological description lacks transparency.

**Response:** The Methods section has been expanded substantially. We now include:

- Databases searched (PubMed, Scopus, AJOL, Google Scholar)
- Search timeframe (2005–2024)
- Detailed inclusion/exclusion criteria
- Screening and coding procedures
- Reference to ENTREQ and PRISMA-ScR reporting standards
- A new *Table 1* summarizing the reviewed studies (country, focus, outcome, and coded attribute).

**Comment 3:** Engage with recent Sub-Saharan Africa (SSA) telehealth literature.

**Response:** We have updated the literature base (2019–2024) to include recent studies from Ghana, Nigeria, Kenya, South Africa, and WHO Africa digital health reports (lines 160–260 and updated references).

**Comment 4:** Strengthen discussion of evidence limitations.

**Response:** We have added a dedicated *Limitations* subsection acknowledging that the synthesis is conceptual and based on secondary sources with limited empirical data.

**Comment 5:** Minor grammatical and stylistic edits.

**Response:** We have carefully edited the manuscript for grammar, clarity, and flow.

**Reviewer #2**

**Comment:** The paper is clear and accessible; findings are relevant and well-organized.

**Response:** We appreciate the positive assessment and have retained this structure while improving clarity and academic tone.

**Reviewer #3**

**Comment:** The paper lacks transparent inclusion/exclusion criteria and appears ambiguous between conceptual commentary and narrative review.

**Response:** We have clarified that this paper is a *conceptual analysis* grounded in theory. The Methods section now explicitly lists inclusion/exclusion criteria and data synthesis procedures (lines 90–130).

**Reviewer #4**

**Comment 1:** Enhance methodological transparency with details on search process and number of included studies.

**Response:** Addressed through the expanded Methods section and *Table 1*. We explicitly note databases, timeframes, and criteria for inclusion/exclusion.

**Comment 2:** Deepen critical engagement with evidence.

**Response:** The Discussion now includes a new subsection titled *Critical Appraisal of Evidence*, assessing study quality, effect size variability, and sustainability of telehealth interventions in SSA.

**Comment 3:** Discuss equity and ethical dimensions of digital adoption.

**Response:** A new *Equity and Ethical Considerations* subsection (lines 240–270) highlights issues of digital exclusion among rural women, elderly populations, and low-literacy groups, as well as privacy and data governance concerns.

**Comment 4:** Provide concrete policy recommendations.

**Response:** The Conclusion now includes actionable recommendations for ministries of health, digital funders, and regional bodies. These focus on integrating telehealth into UHC frameworks and the African Union Digital Health Strategy (2023).

**Comment 5:** Balance theory with practical examples.

**Response:** The Results section now includes real-world SSA examples (Ghana, Kenya, Nigeria, Uganda, and South Africa) illustrating how the five innovation attributes manifest in practice.

**Comment 6:** Update and expand references.

**Response:** References have been updated (2019–2024) to include recent SSA-focused studies and WHO-AFRO reports, formatted per *PLOS* Vancouver style.

**Comment 7:** Include a Limitations statement.

**Response:** A new *Limitations* section explicitly acknowledges that findings are derived from secondary sources and conceptual analysis.

**Summary of Key Revisions**

| **Reviewer Concern** | **Revision Implemented** |
| --- | --- |
| Lack of clarity on study type | Reframed as conceptual synthesis |
| Incomplete methods | Expanded with databases, timeframe, criteria |
| Absence of summary table | Added *Table 1* |
| Limited SSA evidence | Updated references (2019–2024) |
| Missing equity & ethics | Added new subsection |
| Weak policy implications | Added specific, actionable recommendations |
| Lack of limitations | Added dedicated section |
| Minor style issues | Grammar and flow improved |

We believe these comprehensive revisions have substantially improved the manuscript’s clarity, rigor, and relevance. We are deeply grateful for the reviewers’ guidance and the opportunity to resubmit this enhanced version.

Sincerely,

**Delight Tsogbe (Corresponding Author)**

On behalf of all co-authors

Department of Population and Behavioral Sciences

University of Health and Allied Sciences, Ho, Ghana

Email: [delighttsogbe@gmail.com](mailto:delighttsogbe@gmail.com)
